# Editorial: Recent advances in the design and preclinical evaluation of ventricular assist devices

**DOI:** 10.3389/fphys.2023.1322077

**Published:** 2023-11-13

**Authors:** Peng Wu, Zhongjun J. Wu, Haibo Chen, Zengsheng Chen, Xiwen Zhang, Ming Yang

**Affiliations:** ^1^ Jiangsu Key Laboratory for Design and Manufacture of Micro-Nano Biomedical Instruments, School of Mechanical Engineering, Southeast University, Nanjing, China; ^2^ Artificial Organ Technology Laboratory, School of Mechanical and Electrical Engineering, Soochow University, Suzhou, China; ^3^ Department of Surgery, University of Maryland School of Medicine, Baltimore, MD, United States; ^4^ State Key Laboratory of Cardiovascular Disease, Fuwai Hospital, National Center for Cardiovascular Diseases, Chinese Academy of Medical Sciences and Peking Union Medical College, Beijing, China; ^5^ Key Laboratory of Biomechanics and Mechanobiology (Beihang University), Ministry of Education, Beijing Advanced Innovation Center for Biomedical Engineering, School of Biological Science and Medical Engineering, Beihang University, Beijing, China; ^6^ Applied Mechanics Laboratory, Department of Engineering Mechanics, Tsinghua University, Beijing, China; ^7^ Department of Instrument Science and Engineering, Shanghai Jiao Tong University, Shanghai, China

**Keywords:** ventricular assist devices, blood pumps, blood damage, CFD, in-vitro testing

## 1 Introduction

In recent years, ventricular assist devices (VADs, also known as blood pumps) have gradually replaced heart transplantation to provide circulatory support for patients with heart failure in clinical practice (Kormos et al., 2019). However, after the implantation of these VADs, adverse events related to mechanical blood damage, were often reported and have become a major concern during the development of VADs, limiting their clinical and economic benefits (Birschmann et al., 2014; Gurbel et al., 2018). Further efforts are necessitated to improve the performance of VADs by novel designs and evaluation strategies.

Design of VADs must continue to be improved, to optimize the blood-device interaction to reduce blood damage. Various preclinical evaluation approaches have been employed: *in silico* simulations are flexible and inexpensive; during *in vitro* testing, physical devices can be tested and iteratively improved; *in vivo* animal trials can evaluate device-body interactions. Evaluation strategies, both separately and in combination should continue to be optimized and implemented to maximize efficacy of testing. Thus, this Research Topic aims at collecting recent advances in the design techniques and evaluation strategies of VADs. We are delighted to summarize the contributions from the eight publications in this Editorial Article:

### 1.1 Design and optimization of VADs

In recent years, many efforts have been devoted to the optimization of blood pumps (Zhu et al., 2010; Chen et al., 2013; Wiegmann et al., 2018; Wu et al., 2021). Nonetheless, most studies focused on the design of the impeller of conventional centrifugal and axial blood pumps. The percutaneous ventricular assist devices (pVADs) are advanced treatment with the advantages of rapid implantation, less invasive, low rates of complications, and easy nursing. Yun et al. optimized the outflow structures of a pVAD using experiments and CFD, and proposed four outflow structures considering features such as blades, shape of cage bridges. The results showed that outflow structure with curved bridges and bladeless diffuser has an overall lower level of shear stress and hemolysis.

### 1.2 *In-vitro* bench testing of VADs

*In vitro* hemocompatibility testing is a critical step before clinical trials of blood pumps. Li et al. investigated the hemocompatibility of five extracorporeal blood pumps with heparinized porcine blood at nominal operating conditions (5 L/min, 160 mmHg) and extreme conditions (1 L/min, 290 mmHg). Hemolysis and the degradation of von Willebrand factor (VWF) were evaluated. It was found that blood damage was significantly more severe at extreme conditions. The results also demonstrated superior hemocompatibility of two maglev pumps over blood pumps with mechanical bearings.

*In vitro* bench testing for evaluating hydraulic and blood damage performance of VADs, and their physiological interactions with cardiovascular system is vital during the development of VADs. The mock circulatory loop (MCL) is an *in vitro* experimental system that can provide continuous pulsatile flows and simulate different behaviors of human circulation system. Xu et al. reviewed the applications of MCL for testing prosthetic heart valve, VAD, total artificial heart and intra-aortic balloon pump, as well as the pediatric MCLs. This review also pointed out that MCL should be flexibly modularized and adjusted for testing different types of mechanical circulatory support devices, in combination with more advanced measurement methods. Suction is one of the adverse events during the clinical application of VADs which may cause damage to both the patients and medical device. Rocchi et al. validated a suction module (SM) as a test bench for LVAD suction detection and speed control algorithms. The SM was implemented into a hybrid in vitro-in silico cardiovascular simulator. Results showed that the simulator could reproduce most of the pump waveforms observed *in vivo*, and can be used to investigate suction in different conditions.

### 1.3 *In-silico* evaluation of VADs using CFD

CFD has become an important tool for the design, optimization and evaluation of blood pumps (Huo et al., 2021; Wu et al., 2022). Gao et al. investigated the effect of VADs on the hemodynamics in the ascending aorta. Results showed that vorticity intensity under LVAD was significantly higher than that patients without the support of LVAD and the hemodynamics in the ascending aorta were similar to that of a healthy condition. These findings contribute to a more comprehensive understanding of the clinical efficacy of LVAD. Huang et al. investigated the hemolysis performance of a VAD under different wave speed modulation profiles using CFD, coupled with a cardiovascular lumped parameter model. The results reveal that speed modulations slightly affect the pump hemolysis, and the hemolysis differences between the different speed modulation profiles were insignificant. This study suggests that speed modulations could be a feasible way to improve the flow pulsatility of blood pumps while not increasing hemolysis.

### 1.4 Numerical modeling of hemolysis

Typical hemolysis models consider hemolysis as a power-law function of equivalent stress and exposure time (Heuser and Opitz, 1980; Giersiepen et al., 1990; Zhang et al., 2011; Wu et al., 2019). Nonetheless, stress-based models cannot account for blood damage at cellular level. Thus, hemolysis models accounting for effects at cellular or mesoscopic level are necessitated. Xu et al. employed the coarse-grained hemolysis model at the mesoscopic scale based on the transport dissipative particle dynamics method, to predict hemolysis in an axial blood pump. Results showed that the rate of shear stress is the most critical factor in the hemolysis occurring in the rotor region. Wang et al. proposed a hemolysis model considering the aging of blood cells. The critical value of shear stress for the damage of red blood cells was determined through simulating the movement of a single RBC in a shear flow field at the mesoscale. The model was verified against experimental hemolysis results. The results showed that compared with conventional stress-based hemolysis models, the proposed hemolysis model reduced the error of the hemolysis predictions.
